# Ownership and utilization of LLIN after LLIN distribution campaign in a South Western state of Nigeria

**DOI:** 10.1186/1475-2875-9-S2-P34

**Published:** 2010-10-20

**Authors:** Adebusola O Oyeyemi, Abdul-Gafar Alawode, Remi Sogunro

**Affiliations:** 1 Yakubu Gowon Centre, Abuja, Nigeria

## Background

Malaria is one of the leading causes of morbidity and mortality especially among children less than five years of age in malaria endemic countries including Nigeria. In order to minimize socio economic impact of malaria in the country, the Federal Ministry of Health (FMOH) and Roll Back Malaria (RBM) partners have adopted Scale Up for Impact (SUFI) approach in the revised National Malaria Strategic Plan (NMSP). As part of effort to scale up Long Lasting Insecticidal Net (LLIN) ownership and utilization in the country, the target was to increase LLIN coverage and utilization by 100% and 80% respectively in Ekiti State, one of the Southwestern States of Nigeria.

## Objectives

To distribute LLINs to at least 80% of households in Ekiti State with a household having at least two LLINs by 2010 so that at least 80% of those at risk for malaria, particularly children under age five and pregnant women, sleep under insecticide-treated nets.

To evaluate the number of household who received and owned LLINs through the mass campaign and how many of these households were using the LLINs

## Methodology

A stratified random sampling technique was used in selecting the households for the survey and questionnaires were administered by trained interviewers. A total of 2560 HHs were sampled from all the 16 LGAs in the State.

## Results

It was revealed that of the 96.1% of households that received net card during household mobilization, 99.3% redeemed their LLINs, while 5,987 settlements were covered. Proportion of households with at least one net increased from 23 % percent before the campaign to 95.2% after the campaign. 87.3% of the LLINs received were present in the households during the survey and 52.1% of households hung their LLINs. Utilization of LLIN was high among pregnant women and children under five; 88.6% and 69.6% respectively. Utilization rate of LLIN among the sampled population was 59% the previous night before the survey.

## Conclusion

Distribution of LLIN through campaign increased ownership and utilization of LLIN rapidly especially among vulnerable groups; it is possible to reach desired ownership target through a well planed LLIN distribution campaign. However, utilization still fell short of national target especially among non pregnant adult. Additional strategies are needed to increase utilization and sustain current ownership level.

